# Investigation of the impact of pressurization coupled with eccentric resistance training on the explosive strength of the lower limbs in male college students specializing in sprinting

**DOI:** 10.3389/fphys.2026.1758850

**Published:** 2026-07-29

**Authors:** Mengqi Liu, Ruipeng Zhang, Xiaocong Yan, Wenyan Zhao, Keyi Wang

**Affiliations:** 1 Experimental Center for Athletic Performance Development and Evaluation, Harbin Institute of Physical Education, Graduate School, Heilongjiang, China; 2 Harbin Institute of Physical Education, School of Sports Science and Health, Heilongjiang, China; 3 Harbin Engineering University, College of Electromechanical Engineering, Heilongjiang, China

**Keywords:** eccentric training, lower body explosive power, pressurized training, resistance training, sprinting

## Abstract

This study examined the impact of eccentric exercise (ECC) training combined with blood flow restriction training (BFRT) on explosive power in the lower extremities. Sixteen collegiate sprinters were randomly allocated into two groups: the BFRT group, comprising eight male participants, and the ECC group, also consisting of eight men. Participants in the ECC group engaged in eccentric exercises thrice weekly for a duration of six weeks. Alongside the ECC training, participants in the BFRT group had a pressure cuff positioned on the proximal thigh, sustaining a pressure of 150 mmHg. Assessments were performed before and after training to examine hip joint strength, physical fitness (assessed through a 30-meter sprint and vertical jump), anaerobic capacity, and lower limb circumference. The findings indicated that both the ECC and BFRT groups displayed notable enhancements in the isokinetic strength of the hip extensors, with the BFRT group achieving superior results relative to the ECC group. Moreover, BFRT was observed to improve muscle strength on the non-dominant side, consequently diminishing strength asymmetry between both sides. Both training methods enhanced the leaping ability and speed of the collegiate sprinters; however, the BFRT group demonstrated superior efficacy in improving jumping performance. Furthermore, both groups enhanced anaerobic ability and the limb circumference of the lower limb muscles, with BFRT demonstrating more significant advantages than ECC alone. The data indicate that the regular integration of ECC training with BFRT results in superior improvements in lower limb explosiveness, anaerobic capacity, and muscular circumference compared to ECC training performed alone.

## 1 Introduction

The combination of blood flow restriction (BFRT) and eccentric resistance training, hereafter referred to as BFRT-ECC, has emerged as a novel strategy for enhancing explosive power and inducing muscle hypertrophy, garnering significant attention and debate within the sports science community in recent years. Initial evidence suggests that pressure training can markedly improve the adaptive response of muscles, resulting in enhanced muscle explosiveness and increased muscle volume through the constriction of local blood flow (Scarpelli et al., 2021; Wang and Lu, 2022). Nevertheless, certain disputes and unanswered problems persist about the incorporation of training designed to improve explosiveness and facilitate muscle hypertrophy.

LaStayo’s (LaStayo et al., 2003) advanced framework introduces the concept of eccentric training (ECC), asserting that muscle function during eccentric movements is defined by two key mechanisms: the expenditure of energy to decelerate body motion and the accumulation of elastic potential energy for future concentric contractions. The energy expenditure linked to this stretching process is rather low (Julian et al., 2018), however it has the capacity to produce significant muscle strength (Douglas et al., 2017). As a result, eccentric training is receiving heightened interest in the field of strength training. Unlike conventional high-intensity strength training, which often focuses on concentric contractions, eccentric training primarily stresses the eccentric contraction phase of the muscles. This phase entails the muscles opposing external forces while experiencing elongation (Sarto et al., 2020). For example, with the progressive reduction of weights, the muscles perform eccentric contractions. Such contractions selectively engage higher-order motor units and increase the discharge rate of motor units (Sarto et al., 2020), hence enhancing the involvement of additional motor units in the movement. This recruitment increases power production and facilitates enhancements in strength (Fernandez-Gonzalo et al., 2014), explosiveness (Cook et al., 2013), and agility (Fiorilli et al., 2020), ultimately resulting in improved athletic performance.

Sato Yoshitaka (佐藤義昭, 2007) introduced blood flow restriction training (BFRT), or ischemic training, which involves the temporary occlusion of venous blood flow using a specialized device, such as a pressure cuff, applied to the proximal area of a limb during exercise. The main aim of this training technique is to promote muscle cell growth and improve muscle strength. Studies demonstrate that low-intensity resistance training performed with blood flow restriction can produce training outcomes similar to those of conventional high-load resistance training, despite the reduced intensity, thus minimizing the likelihood of exercise-related injuries (Li et al., 2021). Initial research indicates that BFRT markedly enhances the recruitment and activation of motor units by promoting effective coordination within the neuromuscular system.

This training technique elicits a unique physiological stress response that establishes a localized state of hypoxia and reduced blood flow, thereby enhancing the recruitment of type II muscle fibers and leading to considerable fortification of these fast-twitch fibers (Proppe et al., 2023). The effectiveness of Blood Flow Restriction Training (BFRT) is demonstrated by a notable increase in muscle limb circumference and a significant improvement in strength output (Abe et al., 2005; Pearson and Hussain, 2015; Maciel Batista et al., 2020). Moreover, an extensive literature review indicates that BFRT is effective in enhancing muscle strength across diverse age demographics, encompassing adolescents, adults (Dong et al., 2024), and the elderly (Clarkson et al., 2017). Furthermore, it has demonstrated substantial improvements in maximum muscle strength, lower limb explosive power, and muscle circumference within athletic populations, including football players (Hosseini Kakhak et al., 2022), volleyball players (Pişkin et al., 2024), and gymnasts (Yang et al., 2022).

Blood Flow Restriction Training (BFRT) and Eccentric Contraction Training (ECC) markedly improve strength and diverse physical characteristics. Although several researchers have explored the rehabilitation implications of combining ECC and BFRT, there is a scarcity of studies explicitly focusing on BFRT-ECC among male college students in higher education, especially concerning sprinting events. This study is to investigate the impact of a 6-week BFRT-ECC program that combines eccentric contraction (ECC) and blood flow restriction training (BFRT) on isokinetic strength of the hip joint, physical fitness, anaerobic ability, and lower limb circumference in male collegiate sprinters. A comparable study will also be performed with ECC alone.

## 2 Materials and methods

### 2.1 Participant recruitment

This study recruited 16 volunteers from Harbin Sport University, who were randomly allocated to the ECC group (n = 8) and the BFRT group (n = 8) (McKay et al., 2022). According to the classification of athletic performance by McKay et al. (2022), all the subjects in this study were well-trained male athletes of grade two. All participants were second-tier athletes, and no significant variations were seen in height, weight, age, or years of training among them. The Ethics Committee of Harbin Sport University authorized the design and implementation of this study (2024022), adhering completely to the principles of the Helsinki Declaration. To uphold the ethical standards of the study and safeguard the rights and interests of the participants, all subjects provided written informed consent prior to the experiment. (Refer to **Table 1** for additional details).

**Table 1:** Fundamental data of participants.

Participant characteristics	Group (mean ± standard deviation)			
	BFRT group (n=8)	ECC group (n=8)	*t*	*P*
Height (cm)	179.1 ± 5.15	179.4 ± 5.76	0.101	0.914
Weight (kg)	73.63 ± 10.50	74.13 ± 10.26	0.096	0.925
Age (year)	18.50 ± 0.76	18.63 ± 0.92	0.298	0.770
Training Duration	2.50 ± 0.53	2.75 ± 0.46	1.000	0.334

### 2.2 Protocols for resistance training

This study utilized the BTE Eccentron device to perform “electric continuous eccentric pedaling.” From a biomechanical perspective, this protocol differs from traditional eccentric training, which relies on gravitational loading and is often accompanied by a concentric phase; instead, it employs a closed-chain supine pedaling mechanism in which the pedals actively drive the lower limbs at a predetermined rhythm, forcing the athlete to continuously resist external mechanical forces. This design completely eliminates the interference of concentric contraction, achieving isolated and segregated pure eccentric muscle work, and enables the safe and precise quantification of individual eccentric peak force (EPF). The training load for the ECC group was set according to the official BTE protocol (see **Table 2** for details). The training parameters (content, duration, number of sets, rest intervals, and intensity) for the BFRT group were identical to those of the ECC group, with the sole addition of blood flow restriction pressure. Previous studies typically employed a pressure range of 100 to 200 mmHg (Lehnert et al., [[NoYear]]; Patterson et al., 2019; Martinopoulou et al., 2022; Stastny et al., 2024). To determine a more precise pressure value, this study selected the median of the pressure range commonly used in earlier studies, setting the specific pressure at 150 mmHg for the experiment. During the experiment, a 10-cm-wide pneumatic cuff was applied to the proximal thigh; given the continuous nature of the exercise, this pressure was maintained throughout the training session and released immediately upon completion. Although this study did not measure individualized arterial occlusion pressure (AOP), previous research indicates that wider cuffs (such as the 10-cm cuff used in this study) can achieve effective arterial restriction at absolute pressures significantly lower than those required by narrow cuffs (Patterson et al., 2019). Given the high homogeneity of the participants in this study (all were well-trained male sprinters with similar thigh circumferences), 150 mmHg was considered a safe submaximal stimulus sufficient to induce the expected metabolic stress while avoiding the risk of complete arterial occlusion.

**Table 2:** The 6-week recumbent eccentric stepping training program.

Week	Session	Exercise phase	Volume (sets× duration/reps)	Intensity/target force	Cadence (steps/min)	Rest interval
1	1	Test + Continuous Stepping	Test (6 reps) + 1 × 5 min	100% Max/50% EPF	23	Continuous
	2	Continuous Stepping	1 × 8 min	50% EPF (Range: 40-60%)	23	Continuous
	3	Continuous Stepping	1 × 8 min	50% EPF (Range: 40-60%)	23	Continuous
2	4	Test + Continuous Stepping	Test (6 reps) + 1 × 10 min	100% Max/50% EPF	25	Continuous
	5	Continuous Stepping	1 × 10 min	50% EPF (Range: 40-60%)	25	Continuous
	6	Continuous Stepping	1 × 10 min	50% EPF (Range: 40-60%)	25	Continuous
3	7	Test + Continuous Stepping	Test (6 reps) + 1 × 12 min	100% Max/50% EPF	27	Continuous
	8	Continuous Stepping	1 × 12 min	50% EPF (Range: 40-60%)	27	Continuous
	9	Continuous Stepping	1 × 12 min	50% EPF (Range: 40-60%)	27	Continuous
4	10	Test + Continuous Stepping	Test (6 reps) + 1 × 15 min	100% Max/50% EPF	29	Continuous
	11	Continuous Stepping	1 × 15 min	50% EPF (Range: 40-60%)	29	Continuous
	12	Continuous Stepping	1 × 15 min	50% EPF (Range: 40-60%)	29	Continuous
5	13	Test + Continuous Stepping	Test (6 reps) + 1 × 18 min	100% Max/50% EPF	31	Continuous
	14	Continuous Stepping	1 × 18 min	50% EPF (Range: 40-60%)	31	Continuous
	15	Continuous Stepping	1 × 18 min	50% EPF (Range: 40-60%)	31	Continuous
6	16	Test + Continuous Stepping	Test (6 reps) + 1 × 20 min	100% Max/50% EPF	33	Continuous
	17	Continuous Stepping	1 × 20 min	50% EPF (Range: 40-60%)	33	Continuous
	18	Continuous Stepping	1 × 20 min	50% EPF (Range: 40-60%)	33	Continuous

Exercise: All sessions involved recumbent continuous eccentric stepping using the BTE Eccentric device.

EPF (Eccentric Peak Force): Determined dynamically during the first session of each week (Session 1, 4, 7, 10, 13, 16). After a submaximal warm-up, participants performed a “Dosing Test” consisting of 6 maximal effort repetitions (12 strides). The highest force value for each leg was discarded, and the second-highest values were compared. The lower value (representing the weaker leg) was established as the EPF to avoid overexertion.

Intensity (Target Force): The training target force was set at 50% of the calculated EPF. Participants were instructed to maintain their resistance within a Target Range of ±20% of the Target Force (i.e., 40% to 60% of the EPF) throughout the continuous stepping duration.

### 2.3 Indicators of isokinetic strength testing

The assessment of lower limb strength was performed via the CON-TREX PM and MJ isokinetic strength testing apparatus from Switzerland. The maximum power of the hip flexor and extensor muscle groups was assessed at a velocity of 180°/s, comprising one set of five repetitions. The testing regimen commenced with the dominant side of the hip joint, subsequently followed by the non-dominant side, with a 3-minute break between the two assessments.

### 2.4 Indicators of physical fitness assessment

#### 2.4.1 Countermovement jump

The CMJ (countermovement jump) serves as a crucial metric for assessing the pace of muscular strength production (Martinopoulou et al., 2022). Participants execute vertical jumps while positioned on the vertical jump measurement apparatus TZCS-1. Each participant executes 2 practice leaps and 3 official trials (with a 2-minute interval), and their lower limb muscular explosiveness is evaluated based on the maximum jump height attained throughout the ascent.

#### 2.4.2 Sprint of meters

The testing site is the Sports Development and Assessment Laboratory. A mark is established at the initial point, located 30 meters distant, with a timer positioned at that location. During the examination, the participants will employ a standing start method.

### 2.5 Physiological metrics

#### 2.5.1 Anaerobic capacity assessment

Utilizing the Monark 894E anaerobic power bicycle, which is manufactured in Sweden. Participants must perform a 30-second Wingate test with a resistance coefficient of 0.075. The automated system with the device precisely records and analyses all pertinent data in real time.

#### 2.5.3 Circumference of the lower limb

For all participants, bilateral circumferences of the thigh and calf at their thickest points were recorded. All measurements were obtained immediately after waking in a fasted state to minimize acute physiological fluctuations. Standardized form-fitting attire was worn during all assessments to eliminate measurement errors caused by loose clothing or daily physical activities.

### 2.6 Statistical analyses

In this study, data were analyzed using Excel 2016 and SPSS 27.0. Data are presented as mean ± standard deviation (M ± SD). First, the Shapiro-Wilk test was used to assess whether the data followed a normal distribution. For data that followed a normal distribution, a two-way mixed ANOVA was used to test for the interaction between intervention method and time. If the interaction was significant, a paired t-test was used for within-group before-and-after comparisons, and an independent samples t-test was used for between-group comparisons. If the data did not follow a normal distribution, the Wilcoxon signed-rank test was used for within-group pre-post comparisons, and the Mann-Whitney U test was used for between-group comparisons; all hypothesis tests were two-tailed, with statistical significance set at P< 0.05. Additionally, $\eta_{p}^{2}$ and Hedges’g were used to calculate effect sizes. All hypothesis tests were two-tailed, with a statistical significance level set at P< 0.05.

## 3 Results

### 3.1 Isometric muscle strength index

**Figure 1:**
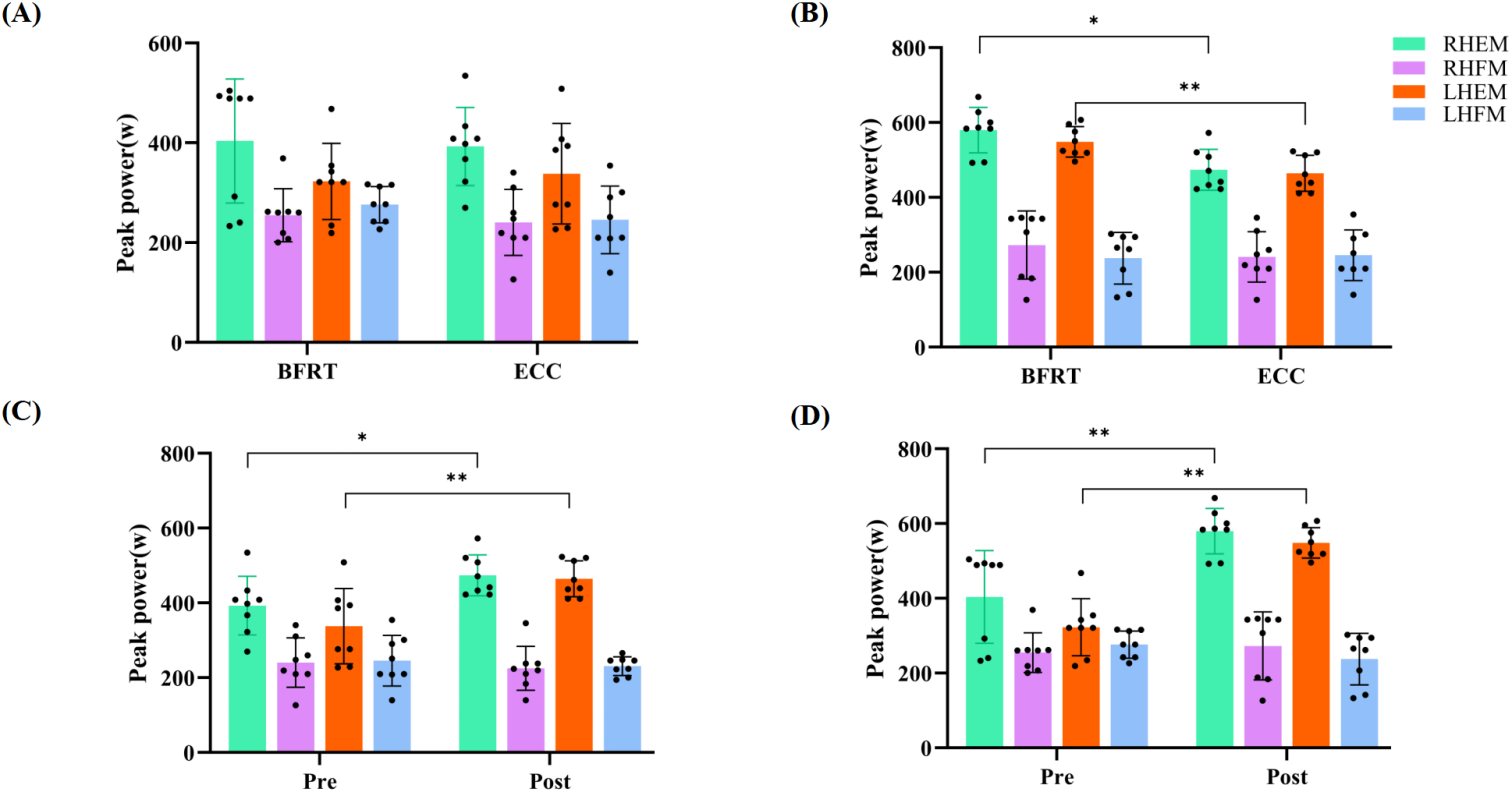
Variations in isometric muscle strength indices prior to and following the experiment. **(A)** Comparison of peak power in the right and left hip flexors and extensors between the two groups before to experimenting. **(B)** Comparison of peak power of right and left hip flexors and extensors between the two groups post-experiment. **(C)** Comparison of peak power between right and left hip flexors and extensors in the ECC group prior to and following the experiment. **(D)** Comparison of peak power between flexor and extensor muscles of the left and right hip in the BRFT group, prior to and following the experiment. * p < 0.05 (significant difference); ** p < 0.01 (highly significant difference).

**Figure 1** illustrates the alterations in isokinetic muscular strength measures prior to and following the experiment. Before the intervention, there were no notable variations in peak power of the left and right hip flexor and extensor muscles between the two groups (P > 0.05) (**Figure 1A**). Findings from a two-way mixed-design ANOVA revealed a statistically significant group × time interaction for peak power of the left and right hip extensor muscles (F(1, 14) = 8.40, P = 0.012, $\eta_{p}^{2}=0.375$; F(1, 14) = 7.90, P = 0.014, $\eta_{p}^{2}=0.361$), indicating that the BFRT group exhibited markedly greater enhancement during the intervention period relative to the ECC-only group. Subsequent within-group *post hoc* analyses indicated that after 6 weeks, both the left and right hip extensor peak power in the ECC group exhibited significant enhancements (P = 0.04, Hedges’ g = 0.78, 95% CI [−0.01, 1.57]; P = 0.04, Hedges’ g = 0.84, 95% CI [0.03, 1.64]) (**Figure 1C**); concurrently, the BFRT group demonstrated a highly significant increase in peak power of both the left and right hip extensors (P = 0.01, Hedges’ g = 1.14, 95% CI [0.25, 2.03]; P< 0.001, Hedges’ g = 1.42, 95% CI [0.44, 2.40]) (**Figure 1D**). Significant variations in right and left hip extensor muscle power were noted between the two groups following the 6-week intervention (P< 0.05) (**Figure 1B**).

### 3.2 Physical fitness index

**Figure 2:**
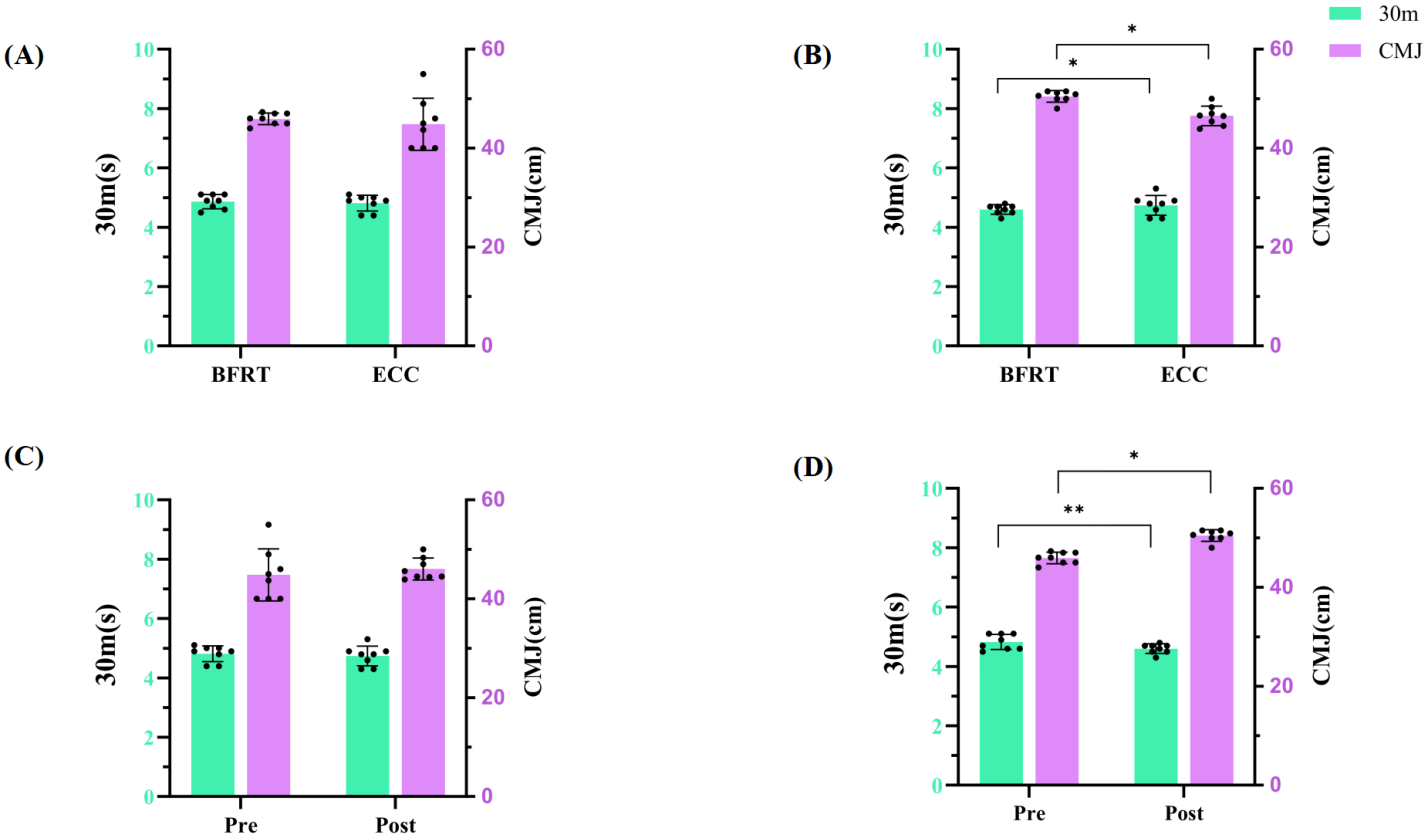
Variations in physical fitness metrics pre- and post-experiment. **(A)** Comparison of physical fitness indices between the two groups prior to and following the experiment. **(B)** Comparison of physical fitness indices between the two groups post-experiment. **(C)** Comparison of physical fitness indices of the ECC group pre- and post-experiment. **(D)** Comparison of physical fitness indices in the BRFT group prior to and following the experiment. * p < 0.05 (significant difference); ** p < 0.01 (highly significant difference).

**Figure 2** illustrates the alterations in physical fitness indicators prior to and subsequent to the trial. Before the intervention, no significant differences were detected between the two groups in the vertical jump (CMJ) and 30-meter sprint (30MS) (P > 0.05) (**Figure 2A**). Results from a two-way mixed-design ANOVA revealed that both CMJ and 30MS displayed a statistically significant group × time interaction (F(1, 14) = 5.99, P = 0.028, $\eta_{p}^{2}=0.300$; F(1, 14) = 7.76, P = 0.015, $\eta_{p}^{2}=0.375$), indicating that the BFRT group exhibited a markedly greater enhancement in explosive power and speed performance during the intervention period relative to the ECC-only group. Subsequent within-group *post hoc* analysis indicated that the ECC group did not demonstrate significant alterations in these two measures following the 6-week intervention (P > 0.05). **Figure 2C** The BFRT group demonstrated statistically significant enhancements in both the CMJ and 30MS (P< 0.001, Hedges’ g = 3.48, 95% CI [1.64, 5.32]; P = 0.02, Hedges’ g = −2.20, 95% CI [−3.48, −0.92]) (**Figure 2D**). Regarding intergroup comparisons, the BFRT group’s performance in the vertical jump (CMJ) and 30-meter sprint (30MS) was considerably superior to that of the ECC group after six weeks (**Figure 2B**).

### 3.3 Physiological metric

#### 3.3.1 Anaerobic capacity

**Figure 3:**
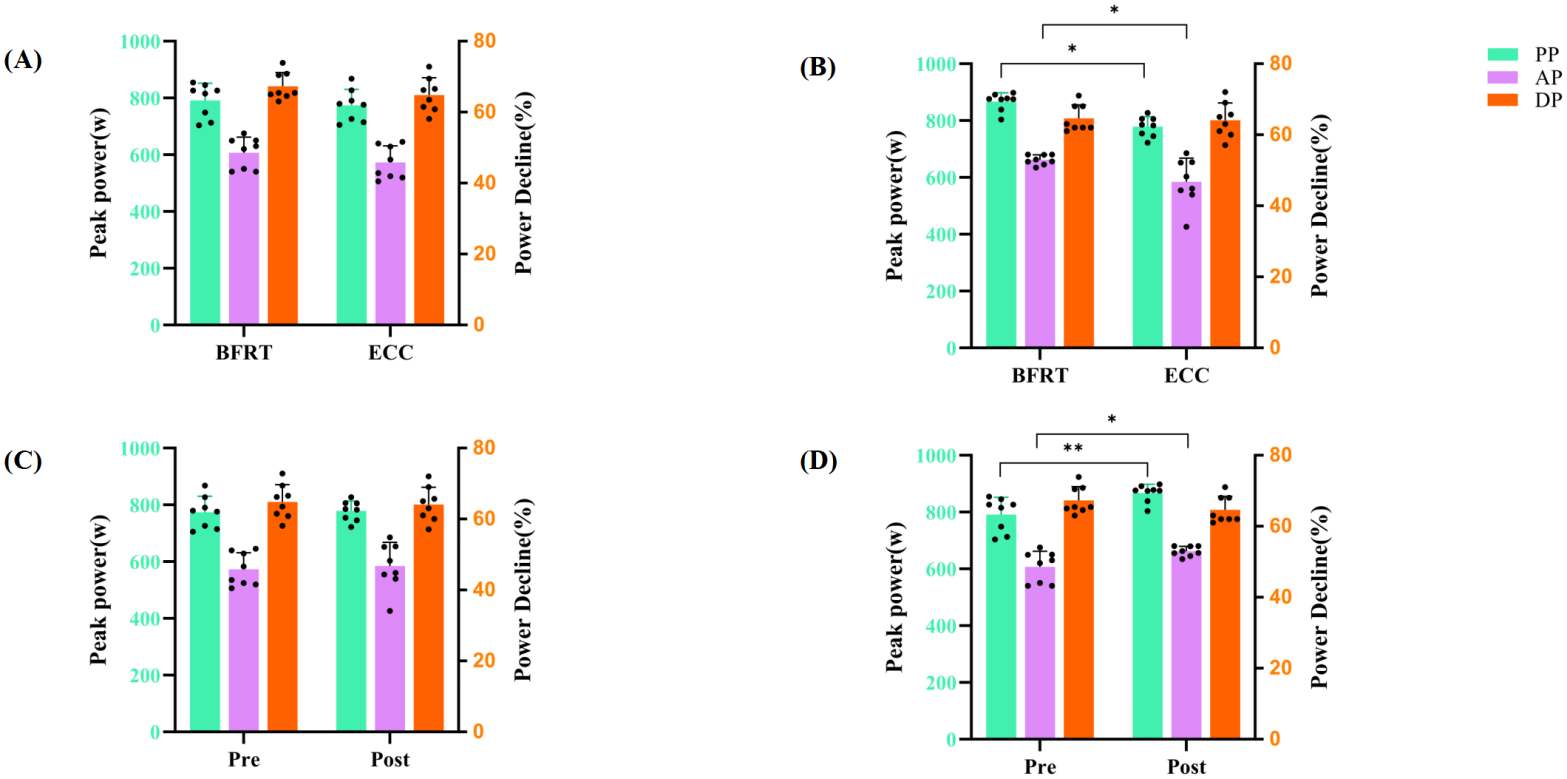
Variations in anaerobic capacity indices pre- and post-experiment. **(A)** Comparison of anaerobic capacity indices between the two groups prior to and following the experiment. **(B)** Comparison of anaerobic capacity indices between the two groups post-experiment. **(C)** Comparison of anaerobic capacity indices in the ECC group prior to and following the experiment. **(D)** Comparison of anaerobic capacity indices in the BRFT group prior to and following the experiment. * p < 0.05 (significant difference); ** p < 0.01 (highly significant difference).

**Figure 3** illustrates the alterations in anaerobic capacity markers prior to and after to the experiment. Before the intervention, there were no notable changes between the two groups in peak power (PP), average power (AP), and power drop rate (PD) (P > 0.05) (**Figure 3A**). Results from a two-way mixed ANOVA revealed that both peak power and average power demonstrated a statistically significant group × time interaction (F(1, 14) = 8.24, P = 0.012, $\eta_{p}^{2}=0.370$; F(1, 14) = 4.80, P = 0.046, $\eta_{p}^{2}=0.255$), indicating that the BFRT group showed a significantly greater enhancement in anaerobic work capacity during the intervention period relative to the ECC group. Subsequent within-group *post hoc* analyses indicated that following the 6-week intervention, the ECC group exhibited no significant alterations in peak power, average power, or anaerobic threshold (P > 0.05) (**Figure 3C**); conversely, the BFRT group showed significant enhancements in both peak power and average power (P = 0.02, Hedges’g = 1.03, 95% CI [0.17, 1.88]; P = 0.03, Hedges’g = 1.14, 95% CI [0.25, 2.03]) (**Figure 3D**). The BFRT group exhibited considerably greater overall effects in peak power and average power than the ECC group after 6 weeks (P< 0.05) (**Figure 3B**).

#### 3.3.2 Circumference of the lower limb

**Figure 4:**
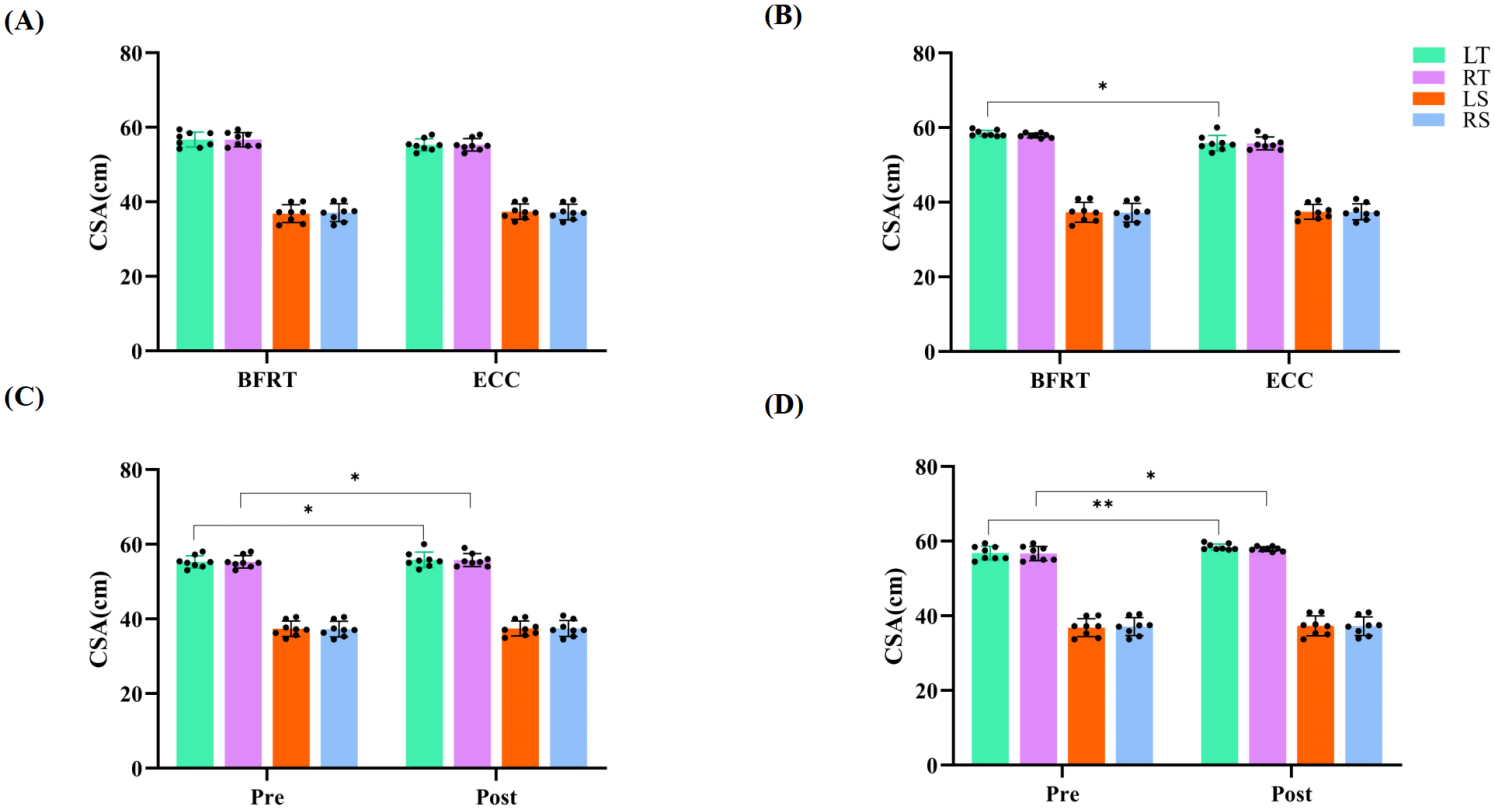
Variations in lower limb circumference indices pre- and post-experiment. **(A)** Comparison of lower limb circumference indices between the two groups pre- and post-experiment. **(B)** Comparison of lower limb circumference indices between the two groups post-experiment. **(C)** Comparison of lower extremity circumference indices in the ECC group pre- and post-experiment. **(D)** Comparison of lower limb girth indices in the BRFT group pre- and post-experiment. * p < 0.05 (significant difference); ** p < 0.01 (highly significant difference).

**Figure 4** illustrates the alterations in lower limb circumference measurements prior to and subsequent to the experiment. Before the intervention, no significant differences were seen between the two groups in the left or right thigh (LT, RT) or the left or right calf (LS, RS) (P > 0.05) (**Figure 4A**). Findings from a two-way mixed ANOVA revealed a statistically significant interaction between group and time for left thigh circumference (F(1, 14) = 5.01, P = 0.042, $\eta_{p}^{2}=0.264$). Conversely, the interaction for right thigh circumference did not achieve statistical significance (F(1, 14) = 1.99, P = 0.180, $\eta_{p}^{2}=0.124$); however, the substantial effect size suggested that the BFRT group demonstrated a pronounced trend of increase. Subsequent within-group analyses indicated that after 6 weeks, both the left and right thighs in the ECC group exhibited significant increases (LT: P = 0.041, Hedges’ g = 0.78, 95% CI [0.06, 1.51]; RT: P = 0.012, Hedges’ g = 1.05, 95% CI [0.25, 1.85]) (**Figure 4C**). Concurrently, the left thigh circumference in the BFRT group demonstrated a highly significant increase (P = 0.007, Hedges’ g = 1.20, 95% CI [0.35, 2.05]), while the right thigh circumference also exhibited a significant increase (P = 0.046, Hedges’ g = 0.76, 95% CI [0.04, 1.48]) (**Figure 4D**). Intergroup comparisons indicated a significant disparity in left thigh circumference between the two groups following 6 weeks of intervention, with the BFRT group demonstrating markedly superior outcomes compared to the ECC group (P< 0.05) (**Figure 4B**).

## 4 Discussion

### 4.1 Effectiveness of integration training on isometric muscular strength

The flexor and extensor muscle groups of a sprinter’s hip are crucial in athletics, as robust hip strength facilitates the effective transmission of generated forces to the lower extremities, thereby enhancing both stride length and frequency, which are vital for augmenting sprinting speed. At an angular velocity of 180°/s, peak power serves as an indicator of the capacity for rapid force production. Following a 6-week experimental intervention, both left and right hip peak power extensors exhibited significant enhancement in the ECC group, while the BFRT group demonstrated a very significant improvement in the same metrics. Lehnert (Lehnert et al., [[NoYear]]) demonstrated that 10 weeks of high-intensity eccentric training in football players led to a substantial enhancement in isometric hip flexors and extensors, a result corroborated by another study indicating that peak moments of 180/s for hip flexors and extensors were significantly elevated compared to a control group following 6 weeks of eccentric training (Stastny et al., 2024). Furthermore, Werasirirat (Werasirirat and Yimlamai, 2022) demonstrated that the average peak moments of the hip flexor and extensor muscle groups in the isometric muscle strength test exhibited a highly significant difference following the initial officer’s participation in a 4-week BFRT intervention.

The findings of the current study align with prior research. This study demonstrated not just an enhancement in flexor strength but also a notably substantial rise in extensor strength. The disparity between anterior and posterior strength was effectively diminished, and the reduction in the gap was remarkable, which is highly significant in reducing hamstring strains. This is closely linked to the eccentric training in the current investigation, which has been shown to markedly enhance the strength of the posterior femoral muscle groups following eccentric training (Gérard et al., 2020). Furthermore, the peak power amplitude of the left hip surpassed that of the right, potentially attributable to the left leg being the nondominant side, which may experience greater stimulation due to under-training (Gonzalo-Skok et al., 2019). Conversely, the constraints on the dominant side’s power lifting indicate a bottleneck in progression within a highly trained state, resulting in superior performance from the nondominant leg. The BFRT group exhibited a more pronounced lifting effect, achieving a somewhat greater lift than the control group. Despite the BFRT group and the ECC group employing identical training content and external loading conditions, the BFRT group exhibited higher training outcomes, potentially attributable to the internal loading mechanism unique to the BFRT group. Conversely, the training advantages of the ECC group were constrained by the absence of similar methods. Notably, the BFRT group achieved a hypoxic and ischaemic environment by implementing a strategy that restricted blood flow during exercise, which markedly intensified the load compared to ECC alone. This process not only enhances the efficient mobilization of fast-twitch muscle fibers (Pazokian et al., 2022) but also initiates a cascade of internal reactions associated with protein synthesis (Wortman et al., 2021), collectively resulting in substantial strength gains, including a marked increase in human growth hormone (HGH) levels and effective suppression of growth-inhibiting hormone (MSTN) (Weaver et al., 2024), both of which work synergistically to significantly promote protein synthesis and expedite strength development (Takarada et al., 2000).

The findings of this study indicated that the impact of the BFRT group on peak hip power surpassed that of the ECC group, and the integration of BFRT and ECC could enhance the efficacy of ECC further. The BFRT group considerably enhanced the strength of the posterior femoral muscle group, mitigated injuries, and positively influenced the improvement of bilateral muscle strength imbalance.

### 4.2 Effectiveness of integrative training on physical fitness quality

The 30-meter run is a crucial component of sprinters’ preparation, significantly enhancing athletes’ starting speed and acceleration. Furthermore, the Countermovement Jump (CMJ) serves as an augmentative exercise that efficiently improves athletes’ lower limb explosive power and coordination (Lockie et al., 2011; Popowczak et al., 2019). Short-distance acceleration and leaping capability are essential in sprinting. The BFRT group had a statistically significant enhancement in the longitudinal jump and 30-meter acceleration run during the physical fitness assessment (P< 0.01). A notable enhancement in vertical jump capacity (Doma et al., 2020; Davids et al., 2021) and speed (Behringer et al., 2017; Wang et al., 2023) has been examined after several weeks of BFRT intervention. The findings of this study align well with prior studies, indicating that the BFRT group exhibited enhancements in both vertical and horizontal leaps, as well as running speed. The notable enhancement in the 30-meter sprint performance seen in the BFRT group is directly associated with particular biomechanical and physiological adaptations of the hip extensors. In the acceleration phase of a sprint, optimizing horizontal impulse is essential, and this biomechanical necessity predominantly depends on the swift power output of the hip extensors (Wiemann and Tidow, 1995; Ishizaka et al., 2019).The simultaneous administration of BFRT and ECC offers an optimal dual synergistic stimulation to fulfil this criterion. ECC training promotes morphological changes, including muscle bundle elongation, enhancing rapid muscle contraction; conversely, the pronounced local hypoxic conditions induced by BFRT compel the earlier and more sustained recruitment of high-threshold Type II muscle fibers (Percival et al., 2022). The integration of brain activation with muscle architecture significantly improves the fast power production potential of the hip extensors. As a result, sprinters can produce enhanced horizontal ground response forces during the limited ground contact period of the acceleration phase, directly correlating with improved 30-meter sprint performance. Nevertheless, research has demonstrated that short-term blood flow restriction training (BFRT) dramatically augments muscle circumference and improves muscle strength. Nonetheless, no statistically significant changes were detected in the enhancements of vertical leaping ability and locomotor speed (Madarame et al., 2011; Fostiak et al., 2022). This disparity can be ascribed to many critical factors: varying training levels of athletes, distinct training cycle schedules, and fluctuations in stress intensity during the application of BFRT. An upward trend was observed in the ECC group; however, it was not statistically significant (P > 0.05). Some studies indicate, a substantial improvement in jumping ability following many weeks of eccentric training (di Cagno et al., 2020) while others show no significant difference between squat jump (SJ) and counter-movement jump (CMJ) after six weeks of eccentric training (Wirth et al., 2015). This may be attributed to variations in training cycles. The BFRT group had superior enhancement in the longitudinal jump compared to the ECC group. Essentially, BFRT may enhance neural adaptability and improve recruitment efficiency and synchronization (Heitkamp, 2015). This indicates that during explosive actions like vertical jumps, it can operate more effectively and synergistically to generate a higher force output (Alberti et al., 2013; Scott et al., 2014).

This study’s results indicated that while both the BFRT and ECC groups exhibited enhancements in speed and jumping, the BFRT group demonstrated a superior effect on longitudinal jump performance compared to the ECC group. Additionally, the integration of BFRT and ECC could further amplify the training outcomes.

### 4.3 Effectiveness of integration training on physiological indices

#### 4.3.1 Anaerobic capacity

Anaerobic capacity is a crucial element of sprint training, serving as the basis for a sprinter’s competitive performance. It is not solely linked to speed and power, but also to the preservation of technical stability during high-intensity exercise. Following the 6-week intervention, both the BFRT and ECC groups exhibited notable enhancements in anaerobic capacity. A study by Crosby (2014) indicated that undergraduate college students who participated in ECC had significant improvements in anaerobic capacity and strength. Moreover, certain studies have demonstrated that ischaemic activation can significantly augment anaerobic capacity, corroborating earlier findings. The BRFT group exhibited enhanced overall performance in peak power and average anaerobic power relative to the ECC group, underscoring the significant metabolic adaptations achieved with the integrated application of BFRT and ECC for sprint-specific training. This can be ascribed to two primary factors: Firstly, the availability of oxygen markedly affects the order of muscle fiber recruitment. Hypoxia may result in the selective activation and significant recruitment of type II muscle fibers (Takarada et al., 2000; Pearson and Hussain, 2015). Secondly, the accumulation of metabolites due to high pressure (Patterson et al., 2019) effectively activates type III and IV afferent nerve fibers while inhibiting the excitation of alpha motor neuroses, thus postponing the onset of fatigue (Loenneke et al., 2010). Conversely, the enhanced anaerobic capacity in the BFRT group may possibly result from greater efficiency in phosphorylation utilization and oxygen uptake (Lindner et al., 2021; de Mendonca et al., 2022).Moreover, the ischemic/hypoxic conditions resulting from impaired blood circulation and reduced oxygen delivery compel active muscles to depend predominantly on anaerobic glycolysis for ATP regeneration (Brooks et al., 2022). Extended exposure to severe metabolic stress induces an increase of essential glycolytic enzymes and improves intracellular buffering capacity by elevating the expression of monocarboxylate transporters (MCTs), thus facilitating swift lactate removal (Ullah et al., 2006; Khajehlandi and Janbozorgy, 2018). These physiological adjustments are essential for sprinters. Augmented anaerobic glycolytic capacity guarantees a swift ATP supply during the initial push-off phase, while enhanced buffering capacity proficiently postpones local muscle exhaustion induced by hydrogen ion accumulation.

Consequently, both the BFRT and ECC groups exhibited considerable improvements in anaerobic capacity; however, the BFRT group attained superior enhancements through the utilization of specialism pressured loads.

#### 4.3.2 Lower limb circumference

A larger lower body volume is generally linked to increased strength, essential for the explosive power and propulsion necessary for sprinting. Following a 6-week experimental intervention, both the ECC and BFRT groups exhibited substantial increases in the circumferences of the left and right sides. Several studies indicate that blood flow restriction training (BFRT) can improve performance not only in rehabilitative (Jack et al., 2023; Li et al., 2023; Solie et al., 2023) and healthy individuals (Centner et al., 2019; Lopes et al., 2019; Baker et al., 2020) but also in athletic populations, including swimmers (Wang et al., 2023), soccer players (Castilla-López and Romero-Franco, 2023), and road cycling (Tangchaisuriya et al., 2022), all of whom demonstrate substantial improvements.

Furthermore, it has been noted that eccentric training can markedly enhance perimeter (Gérard et al., 2020). This assertion was corroborated by another study, which revealed substantial increases in thickness following 12 weeks of eccentric training (Geremia et al., 2019), consistent with earlier findings. Furthermore, the BFRT group surpassed the ECC group, which can be ascribed to the predominant growth mechanism being the metabolic stress response, including fiber recruitment and the activation of protein synthesis pathways (Yuan et al., 2023). BFRT can specifically engage the mammalian target of rapacity (m Tor) in the regulation of mRNA translation initiation and protein synthesis (Bodine et al., 2001; Kumar et al., 2012).

Significantly, although there was no notable alteration in total circumference, calf circumference exhibited substantial alterations in the BFRT group. Numerous prior investigations have corroborated the phenomena of “effect migration.” For example, a research involving 10 young males subjected to a prolonged compression intervention demonstrated increases in circumference at both the compressed and non-compressed regions (Yasuda et al., 2010). This conclusion was validated in another study, where the integration of BFRT with low-load resistance training produced substantial alterations in size and strength in both the distal muscles (biceps, triceps) and the proximal muscles (chest, shoulders) (Dankel et al., 2016). Nevertheless, the findings of the current study are incongruity with prior comparable research and may be ascribed to varying training cycles, a reduced proportion of localized volume in the calf, and the particular training exercises employed. Moreover, research indicates that the impact of BFRT on size alteration is constrained, with growth occurring solely in regions with limited blood flow and no modifications in unrestricted areas (Sakamaki et al., 2011); hence, further investigations are warranted.

## 5 Conclusion

Both the BFRT and ECC groups can markedly enhance lower limb circumference. Nevertheless, by employing specialized pressurized loads, the BFRT group can augment lower limb circumference more effectively than the ECC group. Moreover, the potential of the pressurized intervention to cause impact migration necessitates additional examination.

## 6 Limitation

While this study provides novel insights into the effects of BFRT-ECC on lower limb explosive power and morphological adaptations in collegiate sprinters, several methodological limitations must be acknowledged. First, regarding the blood flow restriction protocol, an absolute pressure of 150 mmHg was universally applied to all participants in the BFRT group. Recent methodological advancements strongly recommend prescribing BFR pressure relative to an individualized Limb Occlusion Pressure (LOP) to account for interpersonal variations in thigh circumference and resting blood pressure. Consequently, the use of a fixed absolute pressure is a limitation, as it may have resulted in varying degrees of relative ischemic stimulus among the athletes. Second, lower limb circumference was utilized as a practical proxy for morphological changes. However, standard tape measurements are highly susceptible to measurement errors and cannot definitively differentiate between true skeletal muscle hypertrophy, alterations in subcutaneous fat, or acute fluid retention (edema) induced by the BFRT-ECC intervention. Therefore, the present findings regarding increased limb girth should not be over-interpreted as direct evidence of expanded muscle cross-sectional area (CSA). Finally, the sample size of this study was relatively small (n=16) and limited to male sprinters. Future studies should employ individualized LOP protocols, recruit larger cohorts, and utilize advanced imaging modalities—such as ultrasonography or dual-energy X-ray absorptiometry (DXA)—to precisely quantify changes in muscle CSA and further elucidate the structural adaptations of this integrated training approach.

## Data Availability

The raw data supporting the conclusions of this article will be made available by the authors, without undue reservation.
